# Next-Generation Sequencing Analysis of the Within-Host Genetic Diversity of Influenza A(H1N1)pdm09 Viruses in the Upper and Lower Respiratory Tracts of Patients with Severe Influenza

**DOI:** 10.1128/mSphere.01043-20

**Published:** 2021-01-06

**Authors:** Ikuyo Takayama, Binh Gia Nguyen, Co Xuan Dao, Thach The Pham, Tuan Quoc Dang, Phuong Thai Truong, Thanh Van Do, Thuy Thi Phuong Pham, Seiichiro Fujisaki, Takato Odagiri, Hideki Hasegawa, Noriko Nakajima

**Affiliations:** a Influenza Virus Research Center, National Institute of Infectious Diseases, Tokyo, Japan; b Bach Mai Hospital, Hanoi, Vietnam; c NCGM-Bach Mai Hospital Medical Collaboration Center, Hanoi, Vietnam; d Department of Pathology, National Institute of Infectious Diseases, Tokyo, Japan; Mount Sinai School of Medicine

**Keywords:** within-host genetic diversity, H1N1, influenza, D222G/N

## Abstract

The influenza A(H1N1)pdm09 virus emerged in April 2009 with an unusual incidence of severe disease and mortality, and currently circulates as a seasonal influenza virus. Previous studies using consensus viral genome sequencing data have overlooked the viral genomic and phenotypic diversity. Next-generation sequencing (NGS) may instead be used to characterize viral populations in an unbiased manner and to measure within-host genetic diversity. In this study, we used NGS analysis to investigate the within-host genetic diversity of influenza A(H1N1)pdm09 virus in the upper and lower respiratory samples from nine patients who were admitted to the intensive care unit (ICU). A total of 47 amino acid substitution positions were found to differ between the upper and lower respiratory tract samples from all patients. However, the D222G/N substitution in hemagglutinin (HA) protein was the only amino acid substitution common to multiple patients. Furthermore, the substitution was detected only in the six samples from the lower respiratory tract. Therefore, it is important to investigate influenza A(H1N1)pdm09 virus populations using multiple paired samples from the upper and lower respiratory tract to avoid overlooking potentially important substitutions, especially in patients with severe disease.

**IMPORTANCE** The D222G/N substitution in the hemagglutinin (HA) protein of influenza A(H1N1)pdm09 virus has been reported to be associated with disease severity and mortality in numerous previous studies. In the present study, 75% of lower respiratory samples contained heterogeneous influenza populations that carried different amino acids at position 222 of the HA protein, whereas all upper respiratory samples only contained the wild-type 222D. These results suggest the influenza A(H1N1)pdm09 virus has diversified inside the host owing to differences in tissue specificity. In this study, the within-host genetic diversity of influenza A(H1N1)pdm09 virus was investigated for the first time using next-generation sequencing analysis of the viral whole-genome in samples extracted from the upper and lower respiratory tracts of patients with severe disease.

## Abstract (precis)

The D222G/N substitution in the hemagglutinin (HA) protein of influenza A(H1N1)pdm09 virus has been reported to be associated with disease severity and mortality in numerous previous studies. In the present study, 75% of lower respiratory samples contained heterogeneous influenza populations that carried different amino acids at position 222 of the HA protein, whereas all upper respiratory samples only contained the wild-type 222D.

## INTRODUCTION

The influenza virus is a single-stranded negative-sense RNA virus belonging to the *Orthomyxoviridae* family. It causes severe acute respiratory tract infections that result in high annual hospitalization rates, especially in children and the elderly (1, 2). It has also caused worldwide pandemics over the past century. The influenza A virus (IAV) genome consists of eight RNA segments encoding the following proteins: polymerase proteins (PB2, PB1, PA), hemagglutinin (HA) protein, neuraminidase (NA) protein, nucleoprotein (NP), matrix (M1 and M2) proteins, and nonstructural (NS1 and NS2) proteins (3). HA and NA are the major viral surface glycoproteins detected by antibodies. The HA protein is responsible for virus attachment and entry into a host cell, whereas NA protein is essential for virus release after budding and, therefore, for the spread of infection.

In April 2009, the influenza A(H1N1)pdm09 virus [A(H1N1)pdm] emerged in Mexico and the United States and spread rapidly throughout the world (4). Infections with A(H1N1)pdm led to relatively mild disease, with moderate and self-limiting upper respiratory tract illness. However, there was an unusually high incidence of viral pneumonia, severe disease, and death in younger age groups compared to that commonly observed with seasonal influenza (5).

IAVs, like other RNA viruses, exist as heterogeneous populations of closely related genetic variants known as quasispecies (6, 7). Human IAV evolves rapidly on a global scale, with these evolutionary dynamics of IAV being driven by processes that take place within and between infected hosts. Evolution of IAV, primarily through antigenic drift and antigenic shift between infected hosts, has been studied using consensus sequencing data that only represents the dominant nucleotide at each viral genome position (8, 9). Consequently, the use of consensus sequencing data has concealed a large proportion of the viral genotypic and phenotypic diversity occurring within an individual host. Recently, high-throughput sequencing technologies, such as next-generation sequencing (NGS), have allowed unbiased assessments of viral populations and measurements of within-host genetic diversity (10, 11).

The evolution of IAV within hosts can be affected by antigenic selection, antiviral drug treatment, tissue-specific selection, and reassortment (12). Human IAV infects heterogeneous, spatially structured populations of epithelial cells in the human airways. A major variation in regions of the human respiratory tract is the distribution of sialic acid receptors that influenza viruses use to enter host cells. In the human body, sialic acid linked to galactose by an α-2,6 linkage (α2,6-SA) is present in the epithelial cells of the upper respiratory tract, trachea, bronchi, and bronchioles. Alternatively, sialic acid linked to galactose by an α-2,3 linkage (α2,3-SA) is found in epithelial cells of the bronchioles and alveoli (13, 14). Most human IAVs preferentially recognize α2,6-SA. In contrast, avian IAVs preferentially recognize α2,3-SA. This tissue specificity of human and avian IAVs can explain why avian-derived viruses cause severe lower lung infections, such as primary viral pneumonia in humans, and show poor human-to-human transmission (14, 15). Although studies on the within-host evolution of human IAV evaluated by high-throughput sequencing technology have increased in the past years (16), most have focused on the diversity linked to drug treatment (17, 18), associated with chronic infection in immunocompetent hosts (19, 20), and as a result of antigenic selection (21, 22).

In this study, the within-host genetic diversity of A(H1N1)pdm was investigated by NGS analysis of the viruses in the nasopharyngeal swab (NPS) and the tracheal lavage aspirate (TLA) from nine patients who were admitted to the intensive care unit (ICU) and required respiratory management with a ventilator.

## RESULTS

### Study population characteristics.

ICU-admitted patients with A(H1N1)pdm infection at Bach Mai hospital in Hanoi, Vietnam, from February 2016 through May 2017 were enrolled in this study. The characteristics of included patients are shown in Table 1. The patients included six men and three women (nine in total), aged 23 to 70 years old (median age, 45 years). All patients presented with pneumonia as confirmed by chest X-ray and exhibited respiratory distress and hypoxemia, thereby requiring respiratory management with a ventilator in the ICU. A(H1N1)pdm infection was diagnosed by real-time reverse transcription (RT)-PCR assay. Three patients had no underlying conditions. Six patients had PaO_2_/FIO_2_ (P/F) ratios of ≤100, suggesting severe hypoxia and respiratory dysfunction upon admission to the ICU. Five patients died after a median of six hospital days (range: 2 to 11 days).

**TABLE 1:** Patient information

Season	Patient	Sex	Age	Underlying conditions^*a*^	P/F ratio on ICU^*a*^ admission	Days from onset		Total hospital time (days)	Outcome	Virus strain name
						ICU admission	Sampling			
2015/16	Pt1	Male	62	Liver disease	90	0	2	5	Death	A/Vietnam/BM1/2016
	Pt2	Male	45	−	281	4	5	29	Alive	A/Vietnam/BM2/2016
	Pt3	Female	70	Diabetes, hypertension	36	8	11	6	Death	A/Vietnam/BM3/2016
	Pt4	Male	64	Liver disease	302	9	11	11	Death	A/Vietnam/BM4/2016
2016/17	Pt5	Male	50	Hypertension, cardiovascular disease, hematological disease	70	7	7	2	Death^*b*^	A/Vietnam/BM5/2017
	Pt6	Female	23	Pregnancy	234	14	14	11	Death	A/Vietnam/BM6/2017
	Pt7	Female	24	SLE, CKD	64	0	2	6	Alive	A/Vietnam/BM7/2017
	Pt8	Male	40	–	62	6	6	16	Alive	A/Vietnam/BM8/2017
	Pt9	Male	28	–	45	5	5	17	Alive	A/Vietnam/BM9/2017

a SLE, systemic lupus erythematosus; CKD, chronic kidney disease; ICU, intensive care unit; –, unknown or no condition.

b Severe condition, discharged by family.

### Viral load of A(H1N1)pdm and other respiratory pathogens.

The copy number of the A(H1N1)pdm M gene in each respiratory sample was calculated based on the results of a quantitative real-time RT-PCR assay targeting the M gene (Table 2). Viral loads in three patients (Pt4, Pt6, and Pt9) were below the calculation limit. For the other six patients, the median viral load in NPS was 1.96 × 10^5^ copies/ml (range: 2.08 × 10^4^ to 3.82 × 10^8^), and the median viral load in TLA was 3.00 × 10^7^ copies/ml (range: 3.18 × 10^6^ to 1.39 × 10^8^). These results suggest these patients were severely infected with A(H1N1)pdm in the upper respiratory tract as well as in the lower respiratory tract.

**TABLE 2:** Quantification cycle (*C_q_*) values derived from real-time RT-PCR-mediated detection of respiratory pathogens and influenza A(H1N1)pdm09 viral load in respiratory samples

Patient	Specimen^*b*^	A(H1N1)pdm viral load (copies/ml)	Virus^*b*^						Bacteria/fungus			
			H1N1	RV	HCoV strain OC43	HCoV strain 229E	HPIV4	HBoV	K. pneumoniae	S. aureus	S. pneumoniae	P. jirovecii
Pt1	NPS^*a*^	2.04 × 10^5^	30.60	37.99								
	TLA	2.90 × 10^7^	22.89		35.59							
Pt2	NPS^*a*^	1.15 × 10^5^	32.63									
	TLA	3.18 × 10^6^	27.19									
Pt3	NPS^*a*^	2.95 × 10^6^	26.84						37.76	36.20		
	TLA	3.10 × 10^7^	23.38						31.96			
Pt4	NPS^*a*^	<1 × 10^4^	35.65						36.98			
	TLA	<5 × 10^3^	36.25						36.43	36.04		
Pt5	NPS^*a*^	3.82 × 10^8^	19.81					37.45	34.99			37.84
	TLA	1.39 × 10^8^	19.71						23.17			28.86
Pt6	NPS	<5 × 10^3^	36.41	37.99			36.17		37.97			
	TLA	8.80 × 10^4^	30.22									
Pt7	NPS	2.08 × 10^4^	32.16			31.65			36.77			
	TLA	7.05 × 10^7^	24.34									
Pt8	NPS	1.87 × 10^5^	28.91						37.49	35.72		
	TLA	3.90 × 10^6^	24.93						36.74			
Pt9	NPS	<5 × 10^3^	35.82									
	TLA	2.22 × 10^6^	25.63								30.66	

a RNA/DNA extractions of five specimens were performed using 100 μl of the specimen.

b NPS, nasopharyngeal swab; TLA, tracheal lavage aspirate; RV, human rhinovirus; HCoV, human coronavirus; HPIV, human parainfluenza virus, HBoV, human bocavirus.

In addition to A(H1N1)pdm, other respiratory pathogens were identified in eight patients (Table 2). Coinfections with human rhinovirus (RV) (*n* = 2), human coronavirus (HCoV) (*n* = 2), human parainfluenza virus (HPIV) (*n* = 1), human bocavirus (HBoV) (*n* = 1), Klebsiella pneumoniae (*n* = 6), Staphylococcus aureus (*n* = 3), Streptococcus pneumoniae (*n* = 1), and Pneumocystis jirovecii (*n* = 1) were observed. Among these, HCoV was the only virus detected in both NPS and TLA. Out of six patients, Pt5 had the highest concentration of *K. pneumoniae* detected in TLA (TLA-Pt5). The concentrations of A(H1N1)pdm were equal to or higher than those of other pathogens in each patient, suggesting that A(H1N1)pdm was the causative pathogen of the disease.

### Summary of NGS analysis and genetic features of A(H1N1)pdm among the patients.

A total of 18 respiratory samples from 9 patients were analyzed by NGS. The percent coverage of the open reading frame (ORF) of each A(H1N1)pdm gene by the final consensus sequences from all the samples is shown in Table S1 in the supplemental material. Viral genes that were not fully covered are indicated by shades of gray in Table S1. All genes of NPS-Pt9 were not fully covered. Except for NPS-Pt9, the ORFs of M and NS genes were fully covered in all the samples. Of the 18 samples, 6 samples (NPS-Pt1, TLA-Pt1, NPS-Pt5, TLA-Pt7, NPS-Pt8, and TLA-Pt8) had 100% coverage for all genes. For the longer polymerase genes, fewer samples (7 to 13 samples) exhibited 100% coverage.

**FIG 1:**
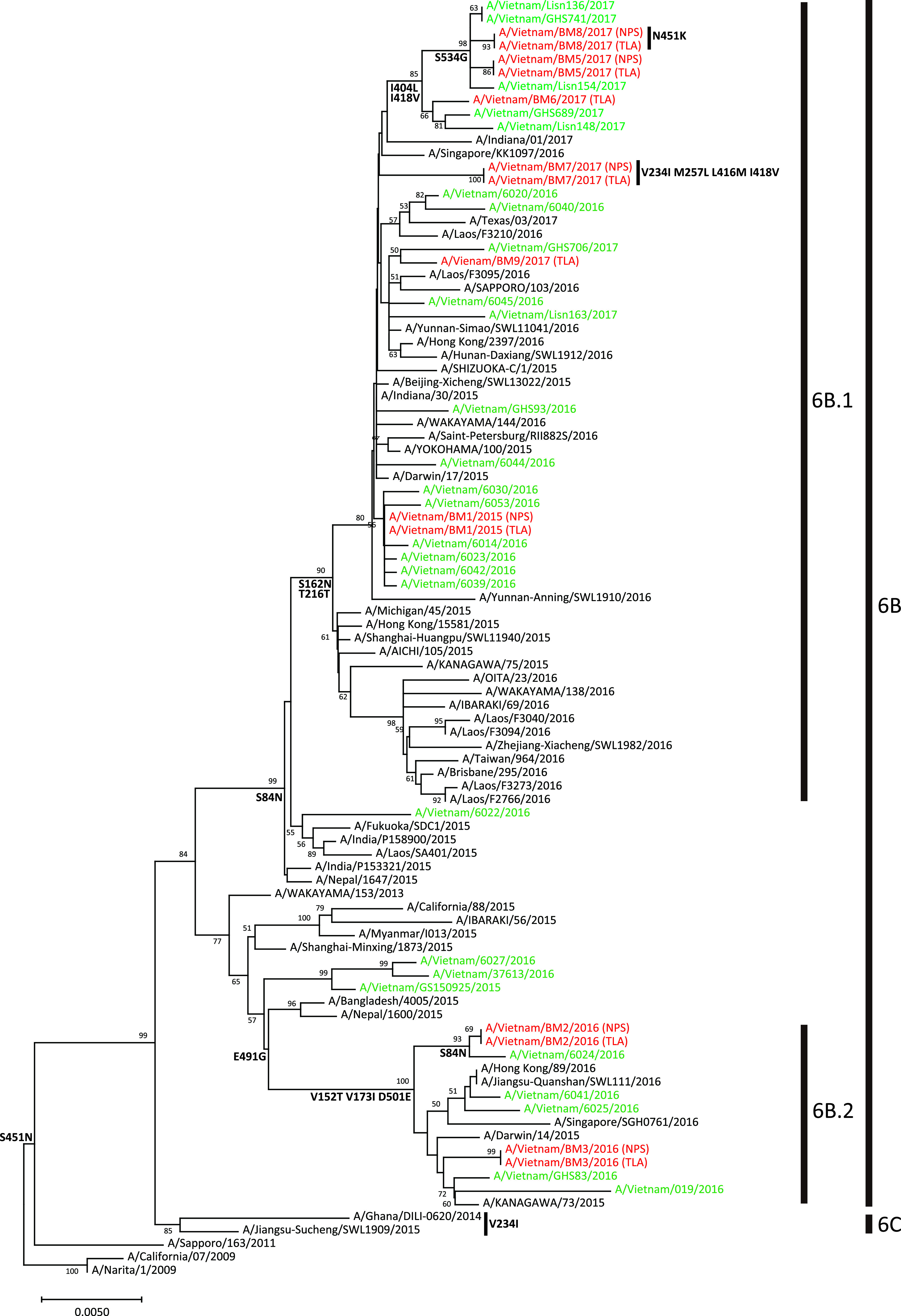
Phylogenetic relationships of the HA gene in influenza A(H1N1)pdm09 viruses. Reference sequences of viruses circulating in Asia in 2015 to 2017 were randomly selected from the GISAID database, and the Vietnamese isolates in 2015 to 2017 are shown in green. The consensus sequences of the A(H1N1)pdm HA genes generated in this study with 100% coverage are shown in red. Bootstrap values were calculated from 1,000 replicates and values higher than 50% are shown next to the branches. The scale bar indicates the nucleotide substitution per site. Fixed amino acid substitutions are mapped at the major nodes of the tree or next to the virus strain name.

Phylogenetic analysis was conducted for the A(H1N1)pdm HA genes that had 100% coverage (i.e., HA genes from NPS-Pt4, TLA-Pt4, NPS-Pt6, and NPS-Pt9 were excluded from the analysis) (Fig. 1). In the 2015/16 influenza season, viruses within the subclade 6B.1 and 6B.2 circulated globally, including in Vietnam (23). As shown in Fig. 1, viruses from Pt1 (A/Vietnam/BM1/2015) belong to clade 6B.1 and viruses from Pt2 and Pt3 (A/Vietnam/BM2/2016 and A/Vietnam/BM3/2016, respectively) belong to clade 6B.2; these results are consistent with the global prevalence of influenza virus in the 2015/16 influenza season. All viruses from Pt5 to Pt9 collected in the 2016/17 influenza season were classified under clade 6B.1, which was globally predominant in that season. Clade 6B.2 is characterized by the amino acid substitutions V152T, V173I, E491G, and D501E (24, 25); viruses from Pt2 and Pt3 possessed these substitutions (Fig. 1). All the amino acid differences between the consensus sequences (including the partially covered consensus sequences) and the consensus sequence of NPS-Pt1 were investigated. Except for the above substitutions characterized in the HA protein of clade 6B.2 and the amino acid substitutions at position 222 of the HA protein, no previously reported amino acid markers were found in any of the proteins of A(H1N1)pdm among the patients (data not shown). Analysis of the amino acid substitution at position 222 of the HA protein is described below as a within-host substitution.

To estimate the genetic diversity of A(H1N1)pdm among the patients, the ratio of nonsynonymous to synonymous substitution rates (dN/dS ratio) averaged over the entire gene of each sample was calculated against the consensus sequence of NPS-Pt1 (Table S1). The dN/dS ratio could not be calculated when either dN or dS was 0. In addition, only consensus sequences with 100% coverage were used for this analysis. For the HA gene, the dN/dS ratios for both NPS and TLA could be determined in five patients (Pt2, Pt3, Pt5, Pt7, and Pt8). Higher ratios were observed in samples classified into clade 6B.1 (Pt5, Pt7, and Pt8) than in samples classified into clade 6B.2 (Pt2 and Pt3) in NPSs and TLAs, respectively. However, the differences were not statistically significant (both *P = *0.2; Mann–Whitney U test).

### Within-host genetic diversity of A(H1N1)pdm.

To investigate the tissue-specific within-host diversity of A(H1N1)pdm, the dN/dS ratios of paired NPS and TLA samples were compared within 33 pairs in 8 viral genes for which the ratio could be determined. The difference between the ratio of NPS and that of TLA was below 0.002 in almost all pairs of samples. In four pairs (NP gene from Pt6 and Pt7, M gene from Pt2, and NS gene from Pt6), the ratio of NPS was 0.005 or higher than the ratio of TLA.

To investigate the detailed population of A(H1N1)pdm within each patient, the differences in the viral protein sequence between NPS and TLA from each patient were analyzed (Table 3). However, amino acid positions that did not have sufficient mapped reads were excluded from this analysis. A total of 47 amino acid substitution positions were found to be different between the paired NPS and TLA samples. Aside from the substitutions at amino acid position 222 of HA protein, which were present in multiple patients, the remaining 46 substitutions were dispersed among the nine patients with each substitution found in only a single patient. The number of amino acid substitutions between NPS and TLA in each patient ranged from 0 to 16; Pt1 did not have any substitutions, whereas Pt7 had 16 substitutions. The correlation between the number of amino acid substitutions between NPS and TLA and the number of days from onset to sampling was low (*R*^2^ = 0.05) (data not shown), which is similar to the results of previous studies (21, 22). In addition, the correlation between the number of amino acid substitutions between NPS and TLA and the number of mapped reads was also low between samples (*R*^2^ = 0.17), as observed in a previous study (19). Notably, there were seven substitution positions for which the frequency of variant amino acids exceeded 50% (Table 3). Among these substitutions, HA-A134T (26) and HA-D222N (27) have been characterized in the HA receptor-binding site. The functions of the other variants have not been reported to date.

**TABLE 3:** Amino acid substitutions of influenza A(H1N1)pdm09 viruses that were different between the nasopharyngeal swab (NPS) and the tracheal lavage aspirate (TLA) samples of each patient

Viral protein^*c*^	Amino acid position	Patient no.	Consensus	Substitution	Frequency of substitution (%)^*c*^	
					NPS	TLA
HA	134	4	A	T	*_–_*^*a*^	100
	222^*b*^					
	406	4	N	S	27.1	–
	471	6	C	R	6.8	–
	492	4	E	G	–	13.5
	500	6	I	V	–	47.6
NA	86	4	A	T	–	100
	145	4	S	P	6.9	–
	184	6	C	R	9.7	–
	255	4	I	T	–	6.0
	344	4	N	D	7.5	–
	431	2	P	L	13.9	–
	436	7	I	V	6.0	–
	452	7	T	A	7.5	–
PB2	82	6	N	I	6.8	–
	227	8	V	A	48.9	–
	314	7	A	T	15.1	–
	401	7	A	D	34.8	–
	547	7	V	I	–	22.3
	724	2	V	G	–	22.8
PB1	7	7	L	P	17.9	–
	42	3	T	K	9.0	–
	62	3	G	D	5.1	–
	190	7	R	S	48.3	–
	388	7	K	R	8.2	–
	615	2	L	P	5.7	–
PA	8	7	C	Y	5.3	–
	99	7	G	R	12.2	–
	220	7	P	S	9.2	–
	697	9	D	G	6.8	–
NP	24	6	E	V	15.0	–
	66	6	M	I	6.3	–
	146	6	A	T	100	46.6
	223	7	C	Y	5.3	–
	329	6	V	L	55.4	–
	360	4	T	A	5.7	–
	384	4	R	G	–	7.3
M1	35	4	K	E	–	9.6
	110	7	H	Y	100	–
	236	2	N	K	100	–
M2	60	7	K	E	5.9	–
NS1	9	6	F	S	18.3	–
	67	4	W	R	–	13.1
	80	7	T	A	–	31.0
	98	6	M	I	77.4	–
NS2	9	6	F	S	18.3	–
	101	6	Q	R	16.7	–

a Variants were not detected at a 5% detection threshold with NGS analysis.

b The variants at codon 222 are shown in Fig. 2.

c HA, hemagglutinin; NA, neuraminidase; NP, nucleoprotein; M, matrix; NS, nonstructural.

### Polymorphisms at amino acid position 222 of the HA protein.

As we observed the within-host population dynamics at position 222 of the HA protein in multiple patients, the ratio of the wild-type and variant amino acids at this position are shown for each sample (Fig. 2). Because sufficient sequence reads could not be mapped for this amino acid position in samples TLA-Pt4, NPS-Pt6, and NPS-Pt9, no data were available for these samples in this region. Although all seven NPS samples only revealed wild-type 222D in the entire viral population, six out of eight (75%) TLA samples had viral populations that carried different amino acids at position 222 of the HA protein. In detail, only Pt9 had a mixture of 222 D/G, whereas the other samples had 222 D/N/G. However, the frequency of 222G in Pt3 and Pt8 and 222N in Pt7 and Pt8 was below 5%, which is the threshold of variant calling in this study.

**FIG 2:**
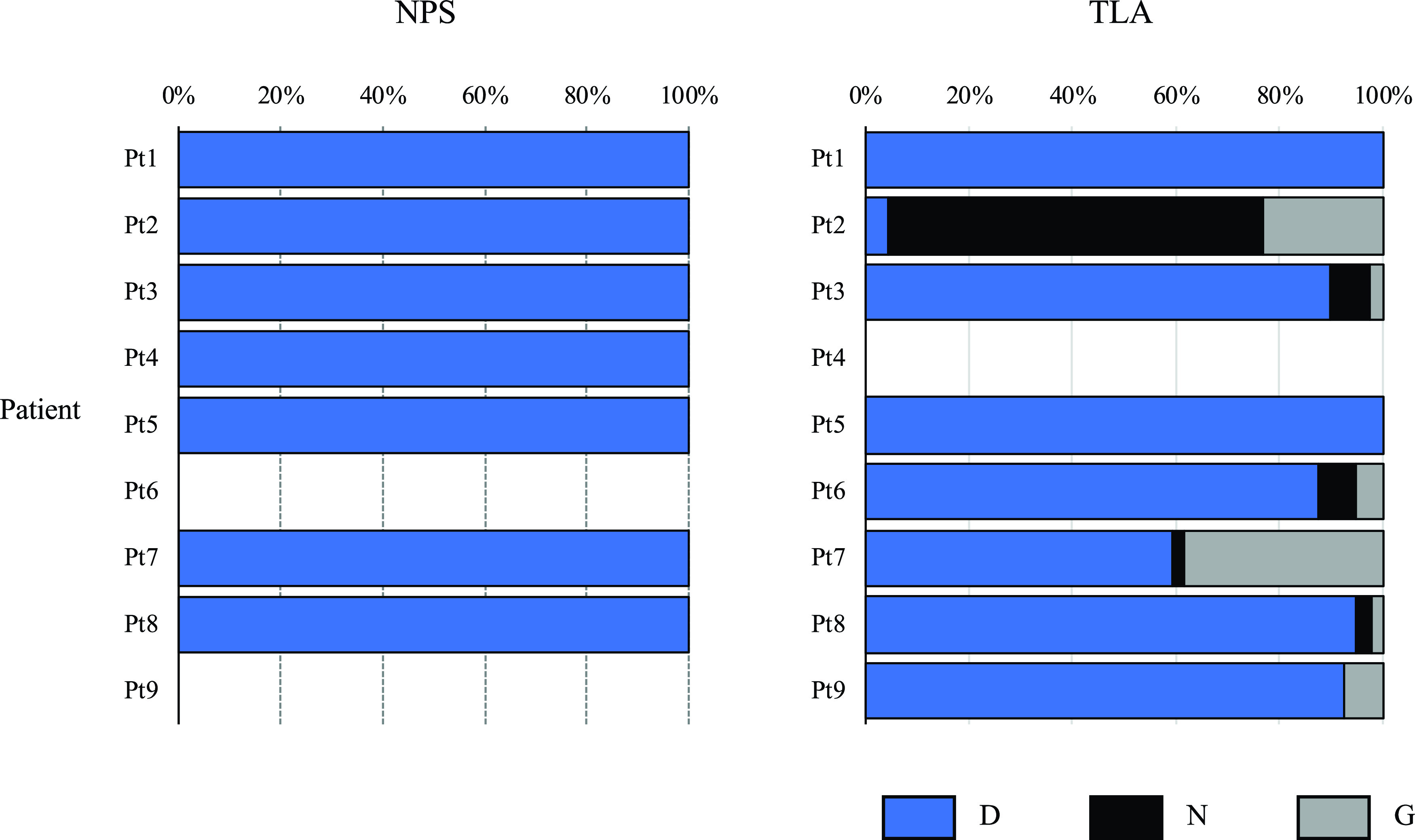
Polymorphisms at amino acid position 222 of the HA protein of influenza A(H1N1)pdm09 virus from nine patients.

Furthermore, we estimated the 47 amino acid differences between NPS and TLA from each viral gene were under positive selection pressure using two selective pressure models: single-likelihood ancestor counting (SLAC) and fixed-effects likelihood (FEL) models (28). Consensus sequences with 100% coverage were used for these analyses. No specific amino acid positions of any viral proteins were detected to be evolving under positive selection using the SLAC model (*P < *0.1). Conversely, amino acids at position 451 of the NP protein and at position 339 of the NA protein were detected to be evolving under positive selection using the FEL model (*P < *0.1). At each position, the amino acid differed between Pt1 and the other patients. The results suggested that the substitutions at amino acid position 222 of the HA protein, as well as the other 46 substitutions, were not under positive selection.

## DISCUSSION

IAVs replicate as quasispecies within the host and are known for their high evolutionary rate. Most within-host diversity is lost upon transmission, but a few infectious viral populations initially escape the mucus of the new host (22, 29). Understanding within-host evolution is important for determining the evolutionary and epidemiological factors that shape the global evolution of influenza virus. In this study, we quantified and evaluated the within-host genetic diversity of A(H1N1)pdm using NGS analysis of the viral whole-genome sequences within NPS and TLA samples from nine patients with severe influenza infection who were admitted to the ICU, requiring respiratory management with a ventilator. The consensus sequences of all viral genes with 100% coverage could be determined in 6 of the 18 samples (NPS-Pt1, TLA-Pt1, NPS-Pt5, TLA-Pt7, NPS-Pt8, and TLA-Pt8) (Table S1). Alternatively, no or partial viral RNA sequences were determined in the remaining samples, possibly owing to low viral load. As the correlation between the number of amino acid substitutions between NPS and TLA samples and the number of mapped reads was low between samples (*R*^2^ = 0.17), the NGS analysis results did not appear to be affected by the bias of viral concentration in each sample. A(H1N1)pdm variants among the patients were classified into clade 6B.1 or 6B.2 based on the HA gene genetic clade, with the results being consistent with the global prevalence of A(H1N1)pdm in the 2015/16 and 2016/17 influenza seasons (Fig. 1). In the analysis of the dN/dS ratio averaged over the entire viral gene to estimate genetic diversity, higher ratios in the HA gene were observed in samples classified into clade 6B.1 (Pt5, Pt7, and Pt8) than in samples classified into clade 6B.2 (Pt2 and Pt3) in NPS and TLA samples, respectively, although the ratio differences were not statistically significant. A previous study has also reported that the dN/dS ratios of the HA gene have changed over influenza seasons (30).

Next, to investigate the tissue-specific within-host diversity of A(H1N1)pdm, we compared the dN/dS ratios of NPS and TLA pairs for 33 pairs of all viral genes for which the ratio could be determined. In four pairs (NP gene from Pt6 and Pt7, M gene from Pt2, and NS gene from Pt6), the ratio of NPS was 0.005 or higher than the ratio of TLA. In a previous study, the dN/dS ratio of the NA gene in left lung clones was reported to be lower than that of the NPS sample in one patient (31). From this result, the authors suggested that negative selection preferentially contributed to the within-host genetic diversity of A(H1N1)pdm in the lower respiratory tract. In the present study, preference for negative selection in the lower respiratory tracts was observed in NP, M, and NS genes in a few samples. However, whether negative selection contributed to the diversity of the NP, M, and NS genes of A(H1N1)pdm in the lower respiratory tracts, rather than in the upper respiratory tracts, was not clarified in this study and should be verified using more samples.

The differences in viral protein sequences between paired NPS and TLA samples in each patient were also investigated (Table 3). A total of 47 amino acid substitution positions were identified. Among these, multiple patients had substitutions at amino acid position 222 of the HA protein, whereas the remaining 46 positions were each only substituted in a single patient. It thus seems these 46 substitutions appeared randomly, whereas the HA-D222G/N substitution may have occurred owing to tissue-specific virus diversity. The HA-D222G/N substitution has been reported in several previous studies. Some studies do not support the association of D222G/N substitution with severe disease, because this substitution is detected even in mild outpatient cases (32). In contrast, numerous other studies have associated HA-D222G with disease severity as this substitution has been described in Europe, the United States, and other countries with a significant frequency in fatal and severe cases (33–37). In recent studies, the HA-D222G/N substitution has been detected in 30% (4/13) of ICU-admitted patients in Italy (38) and the HA-D222G substitution has been detected in 44% (4/9) of severe cases in Norway (33). In both studies, these substitutions were detected only from lower respiratory tract samples. In the present study, 75% (6/8) of TLA samples showed mixed substitutions at amino acid position 222 of the HA protein, whereas all seven NPS samples only showed the wild-type 222D in the entire viral population (Fig. 2). However, we could not find a clear association between the HA-D222G/N substitution and increased disease severity because it is difficult to collect TLA from patients with mild infection; thus, we could not compare prior results with those from patients with mild disease in this study.

The HA-D222G/N substitution has been mapped to the receptor-binding site of HA protein (27). In previous reports, the HA-D222G substitution resulted in a higher binding intensity to α2,3-SA and a lower binding intensity to α2,6-SA in isolated viruses (39, 40). The HA-D222G/N substitution exists as a minor population, whereas HA-D222G appears in <1.8% of A(H1N1)pdm worldwide (41). Based on these considerations, the results of the present study suggest that A(H1N1)pdm with a small population of HA-222G/N infected the patients, and the viral population with HA-222G/N showed enhanced viral invasion to deeper areas of the respiratory tract by amplifying viral tropism consequent to viral receptor distribution in the respiratory tract.

In the present study, other than the HA-D222G/N substitution, no amino acid substitution previously associated with severity or other viral phenotypic characteristics was identified in the samples. Furthermore, the selective pressures acting on each amino acid difference between paired NPS and TLA samples were assessed using two methods. The results showed the HA-D222G/N, as well as the other 46 substitutions, were not under positive selection. Conversely, previous studies have identified that the HA-D222G/N and other substitutions in the antigenic sites were under positive selection through alignment of HA genes in A(H1N1)pdm from clinical specimens, including lower respiratory samples (42). In the present study, any potential genetic selection of A(H1N1)pdm caused by the viral tissue specificity and stimulation by a mechanical ventilator was not determined. Moreover, owing to the small number of patients analyzed in this study, substitution under positive selection may not be detected.

In the present study, within-host genetic diversity of A(H1N1)pdm was investigated for the first time using NGS analysis on the viral whole genome in the upper and lower respiratory tracts of patients with severe disease. Owing to the small number of samples analyzed and the viral load of some samples being too low for NGS analysis, more than half of the samples failed to yield a fully covered consensus sequence of A(H1N1)pdm. Thus, further investigation with more samples is needed. However, this study demonstrated the presence of genetic diversity between the upper and lower respiratory tracts of patients with severe disease, especially at amino acid position 222 of the HA protein. Therefore, it is important to investigate A(H1N1)pdm populations using multiple specimens, such as upper and lower respiratory tract paired samples, in order to avoid overlooking potentially important substitutions, especially in patients with severe disease.

## MATERIALS AND METHODS

### Patients and clinical specimens.

This study targeted ICU-admitted patients with A(H1N1)pdm infection at Bach Mai hospital in Hanoi, Vietnam. Samples from patients receiving mechanical ventilation were analyzed in this study. From February 2016 through May 2017, both nasopharyngeal swab (NPS) and tracheal lavage aspirate (TLA) samples were collected at the same time point from nine patients within 72 h from ICU admission. NPS samples were collected in 1 ml of universal transport medium (UTM; Copan, Brescia, Italy), and all respiratory samples were frozen at −80°C until use. Clinical information, including age, sex, underlying commodity, clinical course, and P/F ratio was collected.

This study was conducted in accordance with the Declaration of Helsinki and was approved by the institutional medical ethical committees of Bach Mai Hospital and the National Institute of Infectious Diseases, Japan. All specimens were collected after obtaining written informed consent.

### Real-time PCR assay.

Total nucleic acids were extracted using the MagMAX CORE nucleic acid purification kit (Thermo Fisher Scientific, Waltham, MA) using 100 or 200 μl of respiratory samples according to the manufacturer's instructions, with an elution volume of 100 μl. Extracts were tested for 33 pathogens using a LightCycler 480 II real-time PCR system (Roche, Basel, Switzerland) and the FTD respiratory pathogens 33 kit (Fast Track Diagnostics, Esch-sur-Alzette, Luxembourg). This kit consists of multiplex real-time PCR assays for the detection of influenza A, B, and C viruses; influenza A subtype H1 pdm09 virus; respiratory syncytial virus; human parainfluenza virus types 1, 2, 3, and 4; human rhinoviruses; human metapneumovirus; human coronavirus OC43, 229E, NL63, and HKU1; human bocavirus; human adenovirus; human enterovirus; human parechovirus; Mycoplasma pneumoniae; Staphylococcus aureus; Chlamydia pneumoniae; Haemophilus influenzae; Haemophilus influenzae type b; Streptococcus pneumoniae; *Legionella* species; Klebsiella pneumoniae; *Salmonella* species; Moraxella catarrhalis; Bordetella pertussis; and Pneumocystis jirovecii. The number of IAV M gene copies per ml of respiratory sample was determined by quantitative real-time RT-PCR assay, as described previously (43).

### Next-generation sequencing.

Viral RNA was extracted from respiratory samples using the QIAamp viral RNA minikit (Qiagen, Hilden, Germany) or the MagMAX CORE nucleic acid purification kit according to the manufacturer's instructions. All eight viral RNA segments of IAV were simultaneously amplified using the SuperScript III one-step RT-PCR system with platinum *Taq* DNA polymerase (Thermo Fisher Scientific) with two slightly modified universal primers (MBTuni-12v2 [5′-ACG CGT GAT CAG CRA AAG CAG G-3′] and MBTuni-13 [5′-ACG CGT GAT CAG TAG AAA CAA GG-3′]) targeting the highly conserved sequence of viral RNA termini, as reported previously (44). The multisegment RT-PCR (M-RTPCR) products were processed for sequencing libraries using the NEBNext Ultra II DNA library prep kit for Illumina (New England BioLabs, Ipswich, MA). Next-generation sequencing (NGS) was performed using the MiSeq reagent kit v3 (150 cycles) (Illumina, San Diego, CA). Following automated cluster generation in MiSeq, the sequences were processed and genetic sequence reads were obtained as FASTQ files.

### NGS data analysis.

Tentative consensus sequences of each sample were generated using FLUGAS software (version 11, World Fusion, Tokyo, Japan). Trimmed reads of samples for each patient were mapped to the tentative consensus sequences for NPS from each patient or to the tentative consensus sequences for TLA if the consensus sequence for NPS was not obtained, using the CLC Genomics Workbench (version 11, Qiagen). The final consensus sequences were extracted from the mapping results for each segment with every base covered by at least 100 reads. For nucleotide sites with <100 reads, a consensus base was not reported, being instead reported as an “N.” Variant calling was performed at a frequency of ≥5%, covered by a minimum of 100 reads. Throughout the manuscript, amino acid positions of the HA protein are based on H1 numbering without the signal peptide. All raw sequencing reads mapped to the tentative consensus sequences were deposited in the DDBJ Sequence Read Archive (DRA). The final consensus sequences with ≥90% coverage of the ORF of each gene were deposited in the Global Initiative on Sharing All Influenza Data (GISAID) database. For each sample and ORF, the dN/dS ratio averaged over the entire gene was calculated compared to the consensus sequence of NPS-Pt1 using the Synonymous and Nonsynonymous Analysis Program (SNAP) provided by the HIV database web site (https://www.hiv.lanl.gov) based on the method described by Nei and Gojobori (45). Tests for positive selection were conducted using SLAC and FEL methods on the Datamonkey 2.0 server (28, 46). For SLAC and FEL methods, a *P* value less than 0.1 indicated significance.

### Phylogenetic analysis.

Phylogenetic analysis for the HA gene was carried out using Molecular Evolutionary Genetics Analysis software (MEGA, version 7.0) (47). The consensus sequences of A(H1N1)pdm HA genes with 100% coverage generated in this study were analyzed. Evolutionary history was inferred using the neighbor-joining method (48). Evolutionary distances were computed using Kimura’s two-parameter method (49). Bootstrap values of HA genes were calculated from 1,000 replicates (50). The representative sequences used in this study for phylogenetic comparison were obtained from the GISAID database.

### Statistical analysis.

The statistical significance of the dN/dS ratio was calculated using the nonparametric Mann–Whitney U test using R software (version 4.0.2) (51). A *P* value of less than 0.05 indicated statistical significance.

### Data availability.

All raw sequencing reads mapped to the tentative consensus sequences were deposited in the DRA database (DRA009446). The final consensus sequences with ≥ 90% coverage of the ORF of each gene were deposited to the GISAID database under accession numbers EPI_ISL_400648, 400649, 400683 to 400686, 400691, 400694 to 400696, 400698 to 400700, 400705, 400708, 400709, and 400711.

10.1128/mSphere.01043-20.1 TABLE S1 Percent (%) coverage of the ORF and dN/dS ratio for each influenza A(H1N1)pdm09 viral gene from all samples based on the final consensus sequences of NGS. Download Table S1, PDF file, 0.1 MB. Copyright © 2021 Takayama et al. 2021 Takayama et al. This content is distributed under the terms of the Creative Commons Attribution 4.0 International license.
