# Supplementary material for: Next-Generation Sequencing Analysis of the Within-Host Genetic Diversity of Influenza A(H1N1)pdm09 Viruses in the Upper and Lower Respiratory Tracts of Patients with Severe Influenza
Source: mSphere. 2021 Jan 6;6(1):e01043-20. doi: 10.1128/mSphere.01043-20 (PMC7845592; doi:10.1128/mSphere.01043-20)
Supplement: TABLE S1 [file mSphere.01043-20-st001.pdf]

TABLE S1 % coverage of the ORF and dN/dS ratio for each influenza A(H1N1)pdm09 viral gene from all samples based on the final consensus sequences of NGS[a]

Viral gene		Patient																	
		Pt1		Pt2		Pt3		Pt4		Pt5		Pt6		Pt7		Pt8		Pt9	
		NPS	TLA	NPS	TLA	NPS	TLA	NPS	TLA	NPS	TLA	NPS	TLA	NPS	TLA	NPS	TLA	NPS	TLA
HA	% coverage of the ORF for the gene	100	100	100	100	100	100	99.9	67.3	100	100	70.1	100	100	100	100	100	0	100
	dN/dS ratio[b]	-	NA[c]	0.057	0.057	0.061	0.062			0.105	0.105		0.091	0.182	0.182	0.156	0.156		0.058
NA	% coverage of the ORF for the gene	100	100	100	100	100	100	100	92.1	100	100	100	100	100	100	100	100	0	100
	dN/dS ratio	-	NA	0.260	0.261	0.183	0.183	0.265		0.088	0.088	0.158	0.158	0.087	0.087	0.053	0.053		0.259
PB2	% coverage of the ORF for the gene	100	100	100	31.8	100	100	100	0	100	100	100	32.2	100	100	100	100	0	44.0
	dN/dS ratio	-	NA	0.085		0.059	0.059	0.150		0.093	0.093	0.089		0.069	0.070	0.120	0.120		
PB1	% coverage of the ORF for the gene	100	100	97.9	37.3	56.5	66.5	99.4	11.7	100	36.6	0	52.0	100	100	100	100	0	62.8
	dN/dS ratio	-	NA							0.030				NA	NA	0.033	0.033		
PA	% coverage of the ORF for the gene	100	100	99.7	89.3	100	100	97.5	11.2	100	100	13.6	100	99.3	100	100	100	26.3	94.1
	dN/dS ratio	-	NA			0.028	0.028			0.019	0.019		0.047		0.026	0.040	0.040		
NP	% coverage of the ORF for the gene	100	100	100	100	100	100	100	94.7	100	100	100	100	100	100	100	100	0	100
	dN/dS ratio	-	NA	0.084	0.084	0.110	0.110	0.148		0.141	0.141	0.189	0.141	0.189	0.158	0.170	0.170		0.141
M	% coverage of the ORF for the gene	100	100	100	100	100	100	100	100	100	100	100	100	100	100	100	100	0	100
	dN/dS ratio[d]	-	NA	0.146	0.097	0.041	0.041	NA	NA	NA	NA	NA	NA	0.072	NA	NA	NA		NA
NS	% coverage of the ORF for the gene	100	100	100	100	100	100	100	100	100	100	100	100	100	100	100	100	0	100
	dN/dS ratio[e]	-	NA	0.172	0.172	0.289	0.289	NA	NA	0.290	0.290	0.296	0.289	0.192	0.190	0.485	0.485		NA

a. Shades of gray: consensus sequences of viral genes showed partial or no coverage.

b. All dN/dS ratios were calculated compared to the consensus sequence of NPS-Pt1.

c. dN/dS ratio was not calculated because either dN or dS was 0.

d. dN/dS ratio of ORF of M1 protein was calculated.

e. dN/dS ratio of ORF of NS1 protein was calculated.
